# Improvement of lateral property of unidirectional-strengthened CFRP laminates using recycled carbon fiber

**DOI:** 10.1038/s41598-023-40813-2

**Published:** 2023-08-22

**Authors:** Annisa Prita Melinda, Rino Yamamoto, Yuichi Miyasaka, Fengky Satria Yoresta, Yusuke Imai, Yoshiki Sugimoto, Kazuki Nomura, Yukihiro Matsumoto

**Affiliations:** 1 Department of Architecture and Civil Engineering, Toyohashi University of Technology, Hibarigaoka 1-1, Tempaku-cho, Toyohashi, Aichi 441-8580 Japan; 2 Department of Civil Engineering, Faculty of Engineering, Universitas Negeri Padang, West Sumatera , 25713 Indonesia; 3 Department of Forest Products, IPB University, Bogor, 16680 Indonesia; 4 National Institute of Advanced Industrial Science and Technology (AIST), 205, Sakurazaka-4chome, Moriyama-ku, Nagoya, Aichi 463-8560 Japan; 5 Development Section, SOBUE CLAY Co., 2-1-4, Shinfune-cho, Minato-ku, Nagoya, Aichi 455-0071 Japan

**Keywords:** Civil engineering, Composites

## Abstract

The unidirectional carbon fiber reinforced polymer (UD-CFRP) lacks the modulus of elasticity and strength in the lateral direction. This study investigates whether matrix resin with CFRP waste, recycled carbon fiber (rCF), can improve the lateral properties of CFRP. In total, twelve CFRP strips specimen were prefabricated of unidirectional carbon fiber (CF) sheet by hand lay-up (HLU) method and were tested by tensile test and X-ray computed tomography (X-ray CT). Factors such as fiber direction and void distribution significantly affecting its mechanical properties are assessed by X-ray CT inspection. It can be seen that rCF is mixed in a random direction at the position filled with matrix resin without rCF. However, a similar frequency of unimpregnation and voids can be observed in both specimens. Test results showed that experimental values of CFRP laminates with rCF-mixed matrix resin increased compared to the CFRP laminates without rCF. The percentage increase in the lateral tensile strength and modulus of elasticity of the rCFRP compared to the control specimen without rCF is 27.36% and 10.62%, respectively. This study proved that rCF can increase the lateral properties of unidirectional CFRP and shows the effective use of rCF for strengthening material in construction applications.

## Introduction

Carbon fiber reinforced polymer (CFRP) is a composite material that has been increasingly used in construction in recent years. It comprises carbon fiber embedded in a polymer matrix, which gives the material strength and durability. One of the main uses of CFRP in construction is to strengthen and retrofit existing structures such as bridges and buildings. This is done by bonding the CFRP strips, sheets, or laminates to the surface/inside of the structure, which increases its load-carrying capacity and resistance to deformation.

CFRP has two main directions based on the orientation of the fiber: lateral and longitudinal. The fiber orientation affects the material’s mechanical properties in different directions, such as strength and stiffness^1–3^. CFRP composites typically exhibit higher strength and stiffness in the longitudinal direction (along the fiber) than in the lateral direction (across the fiber). This directional dependence of mechanical properties is due to the anisotropic nature of CFRP composites, which means that their properties vary with the orientation of the reinforcing fiber. Due to its anisotropic structure, CFRP has lower lateral strength than longitudinal strength. CFRP consists of carbon fiber oriented in a specific direction and embedded in a polymer matrix. The orientation of the fiber determines the mechanical properties of the material. The fiber in CFRP is oriented in the longitudinal direction, giving the material high strength in this direction. However, the fiber is not oriented in the lateral direction, which results in lower strength in that direction^4^. Previous research has focused on the longitudinal direction of CFRP rather than the lateral direction, as the longitudinal direction has the highest strength and stiffness^5–10^. In order to improve the lateral strength of CFRP, an additional strengthening material known as recycled carbon fiber (rCF) is used in this study.

Moreover, CFRP can also be described as sustainable material construction. CFRP composites are considered sustainable construction materials due to their durability, long service life, and recyclability. CFRP waste, on the other hand, will continue to increase significantly shortly. Using these materials can help reduce the environmental impact of construction projects and promote sustainable building practices. Using rCF as a strengthening material in construction helps reduce waste, conserve resources, and reduce the carbon footprint of construction projects^11–14^. It also offers benefits such as improved structural integrity and increased durability over traditional reinforcement materials. An overview of the different techniques developed to recycle fiber-reinforced polymers is presented^15–19^. Recycled carbon fiber can come in different types, depending on the recycling process and the intended application. Some common types of recycled carbon fiber are chopped fiber, milled fiber, carbon fiber mats, carbon fiber fabrics, carbon fiber-reinforced thermoplastics (CFRTPs), and carbon fiber-reinforced thermosets (CFRTs)^20,21^.

In recent years, there has been growing interest in using recycled carbon fiber in construction to strengthen materials. The increase in the use of CFRP composites has a negative impact in terms of the increase in composite waste. Recycled carbon fiber provides a sustainable and cost-effective solution to this problem while offering significant benefits in strength and durability^22–24^. In construction, recycled carbon fiber can be applied to concrete, cement composite, cement mortar, and self-compacting concrete, improving their resistance to stress and impact^25–27^. As such, recycled carbon fiber is becoming an increasingly popular material for strengthening and reinforcing structures in the construction industry. Some reviews of composite recycling carbon fiber can be found in the above literature, but composite recycling is still a relatively new field.

This study investigates whether matrix resin with milled rCF can improve the mechanical properties of CFRP in the lateral direction. Milled fiber is a reinforcing material made from chopped carbon or glass fiber typically used to enhance the mechanical properties of composite materials. In the context of recycled carbon, milled fiber can be used to reinforce recycled carbon composites, which are made from waste carbon fiber that is repurposed for use in new applications. When recycled carbon fiber are processed into a composite material, it may not have the same mechanical properties as virgin carbon fiber, which can impact the performance and durability of the composite. Adding milled fiber to the recycled carbon composite can reinforce and improve the material’s strength, stiffness, and toughness^28,29^. Milled fiber can be used in various forms, such as short fiber or as powder. The fiber is mixed with resin or other matrix material to create the composite, which can then be molded into the desired shape for a specific application. Adding milled fiber can also help the amount of matrix material needed, making the composite more lightweight and cost-effective^30^.

However, a few studies have discussed the performance of CFRP in the lateral direction. This study presents the mechanical properties of unidirectional carbon fiber in the lateral direction with matrix resin mixed with milled rCF. Additionally, the microstructure of each specimen was inspected by optical microscope and X-ray CT (computed tomography). A synchrotron/X-ray inspection is a method for evaluating the quality of recycled carbon fiber materials^31^. The high-energy X-rays a synchrotron generates can penetrate the material deeply, providing detailed information on its internal structure and composition. This information can be used to assess the quality and integrity of the recycled carbon fiber and identify any defects or damage that may affect its performance. X-ray imaging can inspect composite carbon fiber materials to assess their internal structures and identify defects or damage. This is important in ensuring the structural integrity and safety of composite recycled carbon fiber products. In the case of recycled carbon fiber, synchrotron/X-ray inspection is particularly important for ensuring that the material meets the required specifications for use in construction and other applications. The high-resolution images produced by synchrotron/X-ray inspection can reveal any inhomogeneities, cracks, voids, or other defects that may be present in the recycled material and help to determine its suitability for use. Therefore, the effects of rCF in the matrix resin of CFRP in the lateral direction were studied, both in terms of mechanical properties in the lateral direction and X-ray CT inspection.

## Experimental program

### Materials

This study uses a unidirectional carbon fiber (UD-CF) sheet, UT70-30, with dimensions of 250 mm (Length) × 25 mm (Width) and a number of plies is 5, which is used to increase the fiber content. Carbon fiber sheet is a commercial product of Toray Industries, Inc, Japan. Shaped like a sheet, this material is easy to handle and impregnate with resin. The properties of this material are obtained from the manufacturer^32^. The elastic modulus and tensile strength of UT70-30 are 230 GPa and 3400 MPa, respectively. The milled rCF is used as a strengthening material mixed with E2500S resin (Konishi, Osaka, Japan) product. The E2500 has elastic modulus and Poisson's ratio values of 4.1 GPa and 0.37, respectively. For this purpose, 10% rCF volume content was selected to develop CFRP laminates matrix resin with rCF.

#### Milled rCF

**Figure 1:**
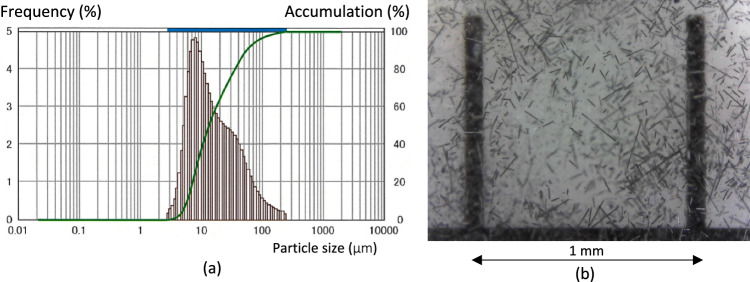
Particle size analysis and microscope view of rCF.

Material milled rCF used in this study was produced by Sobue Clay Co. The raw material (fiber) name of rCF used in this study is intermediate modulus carbon fiber, a commercial product of Toray, called T800S for industrial use. It was experimentally milled by the equipment of Sobue Clay Co. The particle size (fiber length) distribution measurement result can be seen from Fig. 1a. According to the manufacturer, the average length of milled rCF is typically 34.19 μm. The fiber length also can be seen in Fig. 1b, mentioned as a microscope view from rCF. The experimental parameters and volume content compared with weight content between resin and rCF are shown in Table 1.

**Table 1:** Experimental diagram and parameter.

Specimen ID	Size (mm)	rCF volume content	N
HLU_NR	250 × 25	–	5
HLU_rCF	250 × 25	10%	5

### Specimens preparation

A total of twelve specimens, including two specimens for X-ray inspection were tested in this study to investigate the effectiveness of rCF mixing with the resin CFRP laminate in improving the lateral properties of unidirectionally strengthened laminates. The specimen preparation consists of specimen molding for hand lay-up without rCF (HLU_NR) and with rCF (HLU_rCF).

#### Mixing the resin

This study selected 10% rCF volume content to evaluate the fundamental effects of rCF for matrix resin. To mix 10% by volume of rCF into the resin matrix, a weight ratio is used to simplify the calculation. It is known that the matrix density of E2500S resin and milled rCF is 1.15 and 1.8, respectively. Using the ratio of volume to weight, a ratio of 10:90 by volume becomes 15:85 or 17:100 by weight.

#### Specimen molding

**Figure 2:**
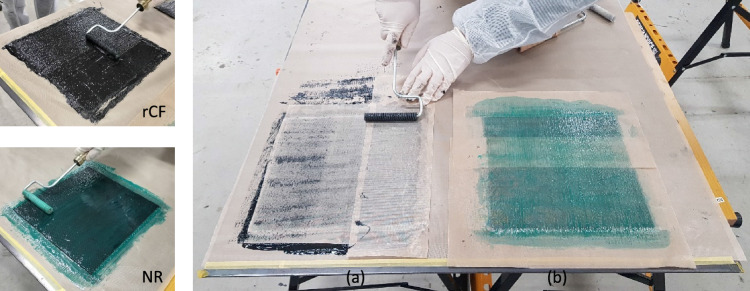
Specimen molding. (**a**) HLU with rCF; (**b**) HLU without rCF (NR).

A total of twelve specimens were made by hand lay-up method. Before cutting each specimen to 250 × 25 mm size, a molding specimen was conducted by 250 × 250 mm size. When molding with a larger size, the CFRP laminates with matrix resin, with or without rCF, aims to spread well. Each carbon fiber sheet is coated with matrix resin mixed with and without rCF. One by one up to the fifth layer, they are coated with matrix resin by hand lay-up as shown in Fig. 2. Figure 2 shows a hand lay-up molding situation with rCF and without rCF (NR). This study selected 10% rCF volume content to evaluate the fundamental effects of rCF for matrix resin. Peel ply covers the top and bottom sides of CFRP and rCFRP. In addition, once the sample was finished, it was important to heat cure the sample to ensure a perfect bond between the carbon fiber, resin and rCF. Finally, after heat curing, the sample is cut to a size of 250 × 25 mm using a water jet cutting machine at the Mikawa Textile Technology Centre.

### Test setup and instrumentation

#### Tensile test of specimens

**Figure 3:**
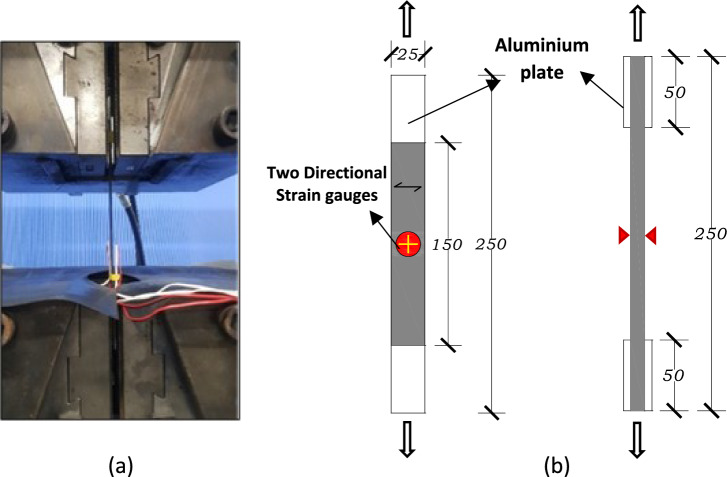
Experimental setup used for the tensile test: (**a**) experimental setup, (**b**) diagram of the experiment.

The mechanical properties in the lateral direction of unidirectional-strengthened CFRP laminates using rCF were investigated experimentally in the tensile test. Tensile tests are carried out to determine the tensile strength, the maximum failure load and lateral modulus of elasticity. The tensile strength of the CFRP laminates was determined using tensile tests in accordance with JIS K 7164^33^, modified from ISO 1268-10: 2005 about specimen geometry and ISO 527-1:2019 about the test method. The tensile tests were conducted using a 1000 kN Maekawa tensile testing machine (Maekawa Testing Machine MFG Co., Ltd., Tokyo, Japan), as shown in Fig. 3a. The load applied during the test was measured by a load cell installed in the machine. Strain gauges were attached to both surfaces of the centre specimens to measure strain values, and a strain rate of 1 mm/min (or 0.04 inch/min) is used for tensile testing. A total of twenty strain gauges were attached to all ten specimens, both strengthened and unstrengthened, to measure strain responses. Two 0/90 direction unified strain gauges are installed on either side of the center of each specimen. The strain gauges used in this study are the FCAB-5 series from Tokyo Sokki Co. Ltd. These values determined each specimen’s modulus of elasticity. At least ten specimens with strain gauge on both sides were tested for each group, as shown in the diagram of experiment Fig. 3b.

#### X-ray CT inspection

CFRP specimens were also analyzed by X-ray CT using a 3D X-ray CT scanner. CFRP and rCFRP specimens were analyzed by X-ray CT using a 3D reconstruction BL8S2 beamline at Aichi Synchrotron Radiation Center. The sample scan area has a cut surface in the 1 mm width. The sample is placed on the tip of the 3 mm diameter pin which is held in place with UV adhesive and placed on the rotating stage. 360° rotation measured in 0.1° increments and measuring time is 15 min. The white X-ray light source was × 10 magnification measurement, the image field of view size is 1.33 × 1.33 mm^2^ with a resolution of 2048 × 2048 pixels and the shooting image pixel size was 0.65 × 0.65μm^2^, respectively.

## Experimental result

### Strength increases

The material properties, including tensile strength, and elastic modulus were obtained from the tensile test. A total of ten specimens with the HLU molding method were divided into two types based on the contained recycled carbon fiber. Five specimens were non-strengthened with rCF, and five others were strengthened with rCF. The tensile test results of CFRP and rCFRP are given in Table 2; maximum load (Pmax [kN]), tensile strength (σmax [N/mm^2^]), and elastic modulus (E [N/mm^2^]). It is clearly shown that the representative strengthening effect by rCF can be confirmed. The percentage increase in the tensile strength and lateral elastic modulus of the HLU rCFRP compared to the control specimen without rCF is 27.36% and 10.62%, respectively. Compared to previous studies on the application of rCF in materials, these results show an effective use of rCF in increasing the tensile strength and lateral modulus of the CFRP. One study investigated the mechanical properties of ultra-high-performance recycled carbon fiber-reinforced concrete and found improved compressive strength, flexural strength, and impact resistance^34^. Another study investigated the mechanical behaviour of carbon fiber and microwave assisted pyrolysis rCF reinforced concrete and found that the addition of 10% carbon fiber to the concrete gave maximum compressive and flexural strength^35^. Additionally, rCF recovered from CFRP by nitric acid showed 1.4 times higher tensile strength compared to virgin carbon fibers^36^. Therefore, the experimental results suggest that recycled carbon fiber can have comparable or even improved material properties compared to carbon fiber without rCF in many construction materials, including CFRP.

**Table 2:** CFRP and rCFRP tensile test result.

Specimen ID	Thickness (mm)	Max load (kN)	Average (N/mm^2^)	Tensile strength (N/mm^2^)	Average (N/mm^2^)	Increase (%)	C.V. (%)	Elastic modulus (MPa)	Average (MPa)	Increase (%)	C.V. (%)
HLU_NR_1	2.28	1.13	1.17	20.40	19.27	–	8.89	8816	6885	–	14.31
HLU_NR_2	2.42	1.07		16.66				6610			
HLU_NR_3	2.48	1.31		21.69				6052			
HLU_NR_4	2.46	1.20		18.44				6367			
HLU_NR_5	2.42	1.13		19.15				6583			
HLU_rCF_1	2.42	1.46	1.60	24.69	24.54	27.36	7.28	8628	7617	10.62	8.79
HLU_rCF_2	2.53	1.86		27.65				7205			
HLU_rCF_3	2.62	1.53		23.93				6875			
HLU_rCF_4	2.57	1.50		22.12				7193			
HLU_rCF_5	2.58	1.66		24.31				8182			

**Figure 4:**
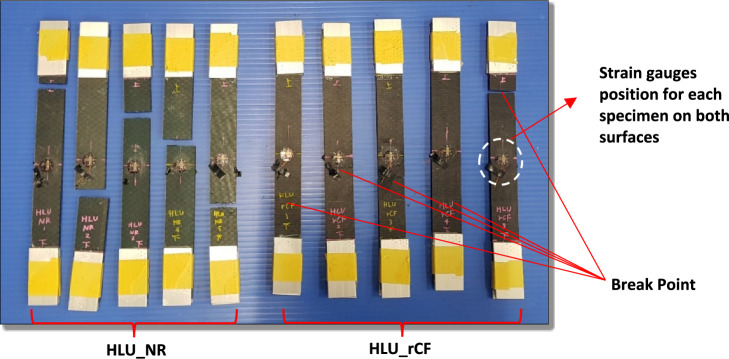
Failure modes after tensile test.

As shown in Fig. 4, a tensile test was carried out on each specimen with and without rCF. The maximum load measurement in this test was evaluated from the load when failure modes occur which correspond to the break point. The elastic modulus was also calculated from the tensile test. The average elastic modulus was taken from 20 to 50% from maximum load that recorded along the tensile test. The least square method is used to get the average elastic modulus value for the average along the range. The average elastic modulus obtained from the physical properties of the CFRP was 6885 MPa for HLU_NR and 7617 MPa for HLU_rCF.

**Figure 5:**
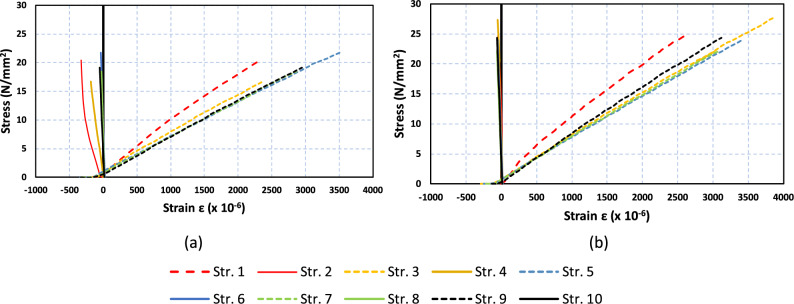
Stress–strain relations of the HLU_NR (**a**) and HLU_rCF specimens (**b**).

Figure 5 shows the stress–strain relations measured from the strain gauges in the experiments. Each figure plots the responses of both non-strengthened and strengthened specimens. The increase in maximum stress for all strengthened specimens is due to the addition of rCF. There were two types of strain gauge directions: 0° and 90°. This figure shows the relationship between each specimen's maximum yield strength and lateral elastic modulus.

The strain values are the average values of the two strain gauges on both sides of the specimens in the exact directions. Elastic modulus is also obtained from the stress–strain curve. Elastic modulus was calculated from the axial stiffness obtained from the experiments and the cross-sectional area of the CFRP/rCFRP tensile test specimen. All dimensions of each specimen, including the widths of the specimens and thicknesses, were measured to calculate the actual cross-sectional areas of all specimens. Using the values of the two-directional strain gauges for the tensile specimens, tensile strength and elastic modulus were calculated for all specimens, as shown in Table 2.

### X-ray CT measurements

**Figure 6:**
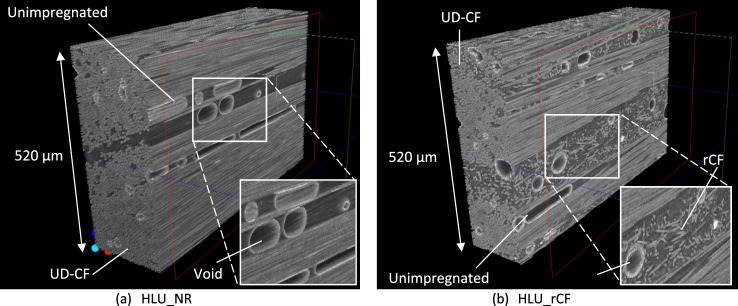
X-ray CT inspection image of specimen.

Factors such as fiber formation and void distribution significantly affecting its mechanical properties are assessed by X-ray CT inspection. Inspected results of the HLU_NR and HLU_rCF specimen by X-ray CT are shown in Fig. 6. A unidirectional carbon fiber (UD-CF) used in this study is clearly shown, for both without and with rCF. The X-ray CT images show the same degree of unimpregnated and voids in both specimens, and the effect of rCF is considered to be small. Obviously, the CFRP specimen without rCF matrix resin shows a lot of voids and non-uniform distribution of matrix resin, which is delivered in the unimpregnated part. Much void spread over the resin layer can be seen in Fig. 6. Comparing both specimens, it can be confirmed that rCF is mixed in a random direction at the position filled with resin without rCF. However, since the same degree of unimpregnated and voids can be seen in both, it is considered that there is no difference in the degree of impregnation due to rCF which is the molding method is the same. There was no significant effect on unimpregnated and voids even if matrix resin with rCF, as shown in Fig. 6b. In addition, further investigation is required to get the reason for not being impregnated with and without rCF, particularly for the molding method and process.

## Conclusions

The results of this study showed that CFRP mixed with rCF can be effectively used to improve the mechanical properties of CFRP in the lateral direction. From the tensile test and X-ray CT the following conclusion were obtained as follows.

- Pre-mixed rCF resin can be used for HLU molding with and without typical voids.
- The mechanical properties of the CFRP with milled rCF show an increase in the tensile strength and modulus of elasticity in the lateral direction. For CFRP with 10% vol milled rCF the increase in tensile strength and modulus of elasticity in the lateral direction is around 27.36% and 10.62%, respectively, although there are still many voids in the specimens with and without rCF.
- X-ray CT images of each specimen show the same degree of unimpregnated and voids. It can be seen in both specimens that the effect of rCF is considered to be small to cover up the void.

## Data Availability

The datasets used and/or analysed during the current study are available from the corresponding author on reasonable request.

## References

[1] Sugimoto Y, Imai Y (2023). Analysis of the in-plane fiber orientation distribution in carbon fiber composites using wide-angle X-ray diffraction. Compos. A Appl. Sci. Manuf..

[2] Özbek Ö, Bozkurt ÖY (2019). The influence of fiber orientation on crashworthiness behavior of carbon fiber reinforced composite pipes. Eur. J. Eng. Sci. Technol..

[3] Özbek Ö, Bulut M, Erkliğ A, Bozkurt ÖY (2022). Interlaminar shear strength and failure analysis of composite laminates with double and triple hybrid configurations. Eng. Struct..

[4] Okuya T, Nakada M, Miyano Y (2013). Reliable test method for tensile strength in longitudinal direction of unidirectional carbon fiber-reinforced plastics. J. Reinf. Plast. Compos..

[5] Al-Furjan MSH (2022). A review on fabrication techniques and tensile properties of glass, carbon, and Kevlar fiber reinforced rolymer composites. J. Market. Res..

[6] Truong GT, Tran HV, Choi KK (2019). Tensile behavior of carbon fiber-reinforced polymer composites incorporating nanomaterials after exposure to elevated temperature. J. Nanomater..

[7] Kumar R, Mikkelsen LP, Lilholt H, Madsen B (2021). Experimental method for tensile testing of unidirectional carbon fibre composites using improved specimen type and data analysis. Materials.

[8] Hernandez DA, Soufen CA, Orlandi MO (2017). Carbon fiber reinforced polymer and epoxy adhesive tensile test failure analysis using scanning electron microscopy. Mater. Res..

[9] Ren X (2022). Effect of corrosion on the tensile and fatigue performance of CFRP strand sheet/steel double strap joints. Eng. Struct..

[10] Zhang X, Shi Y, Li ZX (2019). Experimental study on the tensile behavior of unidirectional and plain weave CFRP laminates under different strain rates. Compos. B Eng..

[11] Ballout W (2022). High performance recycled CFRP composites based on reused carbon fabrics through sustainable mild solvolysis route. Sci. Rep..

[12] Ma J (2022). A novel approach on recycling short-chopped carbon fibers by electric field induced manipulation. Resour. Conserv. Recycl..

[13] Palola S, Laurikainen P, García-Arrieta S, Astorkia EG, Sarlin E (2022). Towards sustainable composite manufacturing with recycled carbon fiber reinforced thermoplastic composites. Polymers.

[14] Pimenta S, Pinho ST (2011). Recycling carbon fibre reinforced polymers for structural applications: Technology review and market outlook. Waste Manag..

[15] Oliveux G, Dandy LO, Leeke GA (2015). Current status of recycling of fibre reinforced polymers: Review of technologies, reuse and resulting properties. Prog. Mater Sci..

[16] Yu K, Shi Q, Dunn ML, Wang T, Qi HJ (2016). Carbon fiber reinforced thermoset composite with near 100% recyclability. Adv. Funct. Mater..

[17] Wang Y (2015). Chemical recycling of carbon fiber reinforced epoxy resin composites via selective cleavage of the carbon-nitrogen bond. ACS Sustain. Chem. Eng..

[18] Okajima I, Hiramatsu M, Shimamura Y, Awaya T, Sako T (2014). Chemical recycling of carbon fiber reinforced plastic using supercritical methanol. J. Supercrit. Fluids.

[19] Okajima I, Sako T (2017). Recycling of carbon fiber-reinforced plastic using supercritical and subcritical fluids. J. Mater. Cycles Waste Manag..

[20] Wang Y, Li AY, Zhang SH, Guo BB, Niu DT (2023). A review on new methods of recycling waste carbon fiber and its application in construction and industry. Constr. Build. Mater..

[21] Tian Z, Wang Y, Hou X (2022). Review of chemical recycling and reuse of carbon fiber reinforced epoxy resin composites. New Carbon Mater..

[22] Sukanto H, Raharjo WW, Ariawan D, Triyono J (2021). Carbon fibers recovery from CFRP recycling process and their usage: A review. IOP Conf. Ser. Mater. Sci. Eng..

[23] Pakdel E, Kashi S, Varley R, Wang X (2021). Recent progress in recycling carbon fibre reinforced composites and dry carbon fibre wastes. Resour. Conserv. Recycl..

[24] Butenegro JA, Bahrami M, Abenojar J, Martínez MÁ (2021). Recent progress in carbon fiber reinforced polymers recycling: A review of recycling methods and reuse of carbon fibers. Materials.

[25] Danish A (2022). Utilization of recycled carbon fiber reinforced polymer in cementitious composites: A critical review. J. Build. Eng..

[26] Xiong C (2021). Sustainable use of recycled carbon fiber reinforced polymer and crumb rubber in concrete: Mechanical properties and ecological evaluation. J. Clean. Prod..

[27] Isa A (2022). A review on recycling of carbon fibres: Methods to reinforce and expected fibre composite degradations. Materials.

[28] Akbar A, Kodur VKR, Liew KM (2021). Microstructural changes and mechanical performance of cement composites reinforced with recycled carbon fibers. Cem. Concr. Compos..

[29] Song W, Magid A, Li D, Lee KY (2020). Application of recycled carbon-fibre-reinforced polymers as reinforcement for epoxy foams. J. Environ. Manag..

[30] Newman B, Creighton C, Henderson LC, Stojcevski F (2022). A review of milled carbon fibres in composite materials. Compos. A Appl. Sci. Manuf..

[31] Sugimoto Y, Shimamoto D, Hotta Y, Niino H (2022). Estimation of the fiber orientation distribution of carbon fiber-reinforced plastics using small-angle X-ray scattering. Carbon Trends.

[32] *Numbering Rules for Torayca*^*TM*^* Fabric Products 3. Standard Packing 4. Handling Precautions for Carbon Fiber*. www.cf-composites.toray.

[33] *JIS K 7164:2005 Plastics—Test Methods for Tensile Properties—Part 4: Test Conditions for Isotropic and Orthotropic Fiber-Reinforced Plastics.*

[34] Patchen A, Young S, Penumadu D (2023). An investigation of mechanical properties of recycled carbon fiber reinforced ultra-high-performance concrete. Materials.

[35] Li YF (2021). An experimental study on mechanical behaviors of carbon fiber and microwave-assisted pyrolysis recycled carbon fiber-reinforced concrete. Sustainability.

[36] Sakai A, Kurniawan W, Kubouchi M (2023). Recycled carbon fibers with improved physical properties recovered from CFRP by nitric acid. Appl. Sci..

